# Molecular Characterization of *Echinococcus granulosus* from Hydatid Cysts Isolated from Human and Animals in Golestan Province, North of Iran

**Published:** 2012

**Authors:** Sh Gholami, M Sosari, M Fakhar, M Sharif, A Daryani, MB Hashemi, M Vahadi

**Affiliations:** 1 Department of Parasitology and Mycology, Faculty of Medicine, Mazandaran University of Medical Sciences, Sari, Iran; 2 Department of Biochemistry and Genetics, Faculty of Medicine, Mazandaran University of Medical Sciences, Sari, Iran; 3 Department of Microbiology, Faculty of Medicine, Mazandaran University of Medical Sciences, Sari, Iran

**Keywords:** Molecular Characterization, *Echinococcus granulosus*, Hydatid cysts, Iran

## Abstract

**Background:**

The aim of the present study was to determine the molecular characteristics of *Echinococcus granulosus* from paraffin-embedded tissues of hydatid cysts isolated from human and protoscoleces of hydatid cysts from sheep, cattle and camel isolates using PCR- RFLP of ITS1- rDNA analysis in Golestan Province, northern Iran.

**Methods:**

*E. granulosus* isolates from human patients infected with hydatid cyst and protoscoleces from hydatid cysts of sheep, cattle and camel isolates were collected from different hospitals and the abattoir throughout the Golestan Province. In all, 60 *E. granulosus* genomic DNA were extracted and examined by PCR - ITS1 of rDNA and amplified using BD1 / 4S and EGF1 / EGR2 primers, followed by RFLP using *Alu1, Msp1* and *TaqI* restriction enzymes.

**Results:**

The PCR-ITS1 products obtained from sheep, cattle and human isolates were similar to sheep strain (1000 bp and 391 bp). Majority of the camel samples yielded 295 bp DNA bands. RFLP -ITS1 of *E. granulosus* with *Taq1* in human, sheep and cattle isolates showed similar patterns in the number and size of DNA. RFLP methods in camel isolates showed a different genotype, using *Taq1*, whereas no DNA bands were observed using *Alu1* in camel and human isolates. Therefore, two clearly distinguishable banding patterns of *E. granulosus* were obtained with the three enzymes, which separating human, sheep and cattle isolates from the camel origin.

**Conclusion:**

The results indicate the possible of transmission of the G1 and G6 genotypes of *E. granulosus* between livestock animals and human in Golestan Province.

## Introduction

Cystic hydatid disease, caused by the metacestode of *Echinococcus granulosus*, is one of the most important serious parasitic diseases in the medical, veterinary sciences and with economic consequences in different regions of Iran and the world (1, 2). *E. granulosus* is made up of several genotypic strain groups in endemic areas with world wide distribution in human and animal. In the past, different isolates of *E. granulosus* were characterized on the basis of differences in morphological characteristics, biochemical composition, isoenzyme profiles, developmental patterns and intermediate host specificity (1, 3, 4,), but more recently, various molecular tools (RFLP, PCR-RFLP and mitochondrial DNA sequences) were used. These techniques proved a reliable and number of workers used these techniques for the identification of genotype/ strain of *E. granulosus*, as well as transmission patterns where strains occur sympatrically (5–7). To date, molecular studies have confirmed the concept of strain diversity in *E. granulosus*, but previous studies were done based on morphological and biological features. Molecular genetic studies have identified 10 different genotypes (G1-G10) within *E. granulosus* till now (7–13). *E. granulosus* has been ordered into *E. granulosus* sensu stricto (G1–G3), *E. equinus* (G4), *E. ortleppi* (G5) and *E. canadensis* (G6–G10) (5, 12–14). However, the status of different strains of *E. granulosus* have been described using various morphological and biological criteria in the past, and more recently, genetic analysis using molecular tools have been reviewed (6, 15–18). A study established a new PCR protocol for detection and intra-specific identification of *E. granulosus* genotypes from human in formalin fixed paraffin-embedded tissues (FFPT) in patients with histologically confirmed echinococcosis as a source of DAN in Austrian hospital (19).

In Iran, cystic echinococcosis (CE) is one of the major parasitic problems seen in both human and livestock animals (8, 9). Hydatidosis has been reported from different parts of Iran where it was found various livestock animals and human to harbor hydatid cysts (20–24). Among various livestock, sheep and camel in Iran were considered as the most common and suitable intermediate hosts for cysts development, because the prevalence and fertility rate of the cysts were found very high in these animals (25–27). In different regions of Iran, three distinct genotypes (G1, G2, and G3) within *E. granulosus* have been identified by molecular analysis where, sheep, camel and buffalo have an important role in transmission cycle of cystic hydatid disease to human (14, 22, 28, 29).

Until now in Iran, different genotypes of *E. granulosus* were identified using molecular tools in human, sheep, cattle, buffalo and camel isolates (12, 23, 28, 30–33), but the parasites materials from paraffin-embedded tissues of hydatid cysts isolates from human were not characterized before, particularly at the endemic areas, especially in north of Iran. However, the sources of infection in humans and the role of intermediate host reservoirs remain to be determined (32, 34). Therefore, identification of different genotypes of parasite could help the control programs of disease, particularly in humans in the endemic areas. Cystic echinococcosis is also a zoonotic infection with economic impact and a threat to public health in Golestan Province, northern Iran, with a wide region of animal husbandry. Therefore, the aim of the present study was to determine the molecular characteristics of *E. granulosus* from paraffin-embedded tissues of hydatid cysts isolated from human and protoscoleces of hydatid cysts from sheep, cattle and camel isolates using PCR- RFLP of ITS1 analysis in Golestan Province.

## Materials and Methods

### Patients

The present study was carried out on formalin fixed paraffin-embedded tissues (FFPT) from 30 patients with histologically confirmed echinococcosis, conducted between 2004 and 2008 in several hospitals, in Golestan Province. All patients were identified as being infected with cystic echinococcosis by histopathologically (detection of PAS- positive laminated layers and /or of protoscoleces and /or hooklets) of the respected tissues. For each patient, 2 thickness sections (6 µm) were prepared from tissue blocks and excess paraffin was trimmed. Sections were placed in 1.5 ml microtubes and deparaffinized with 1 ml xylene for 10 min at 37 °C. Subsequently, samples were centrifuged at 1500 rpm for 5 min and the supernatant was removed. This procedure was repeated. After deparaffinization, rehydration in 100%, 90%, 80% and 70% ethanol was followed (19). Then, 70% ethanol was removed and tissue lysis solution was added for DNA extraction.

### Animal samples

Thirty animal hydatid cyst isolates (15 sheep, 10 cattle and 5 camels) were collected from several abattoirs of Golestan Province, northern Iran. The cysts were processed separately and samples represented protoscoleces, aspirated from an individual hydatid cyst. Then, protoscoleces were rinsed in physiological saline solution, fixed in 95% (v/v) ethanol and stored at -20 °C. For the genomic DNA (g DNA) extraction, the protoscolices were rinsed several times with sterile distilled water to remove the ethanol prior to DNA extraction.

### DNA Extraction

A total of 60 samples were examined for DNA extraction and amplification of ITS1-rDNA. *E. granulosus* genomic DNA (gDNA) from the human and animal isolates was extracted from each cyst sample using DNA extraction kit (Cinnagen; Tehran, Iran) according to manufacturer's instruction. Approximately 1 ml packed volume of protoscolices was mechanically grinded in 180 µl lysis buffer and 20 µl proteinase K was used and incubated at 55 °C for 1-3 hours and terminated with 10 min incubation at 95 °C to inactivate the proteinase K. The pure DNA was eluted in Tris-HCl buffer by effective washing and stored at -20 °C. The concentration of DNA was determined using spectrophotometric method. Finally, sixty samples (30 from human, 15 from sheep, 5 from camel and 10 from cattle isolates) were used for DNA amplification and PCR-RFLP analysis.

### PCR-RFLP analysis

*Echinococcus granulosus* genomic DNA samples were analyzed by polymerase chain reaction (PCR) of rDNA internal transcribed spacer 1 (ITS1-rDNA) and PCR-restriction fragment length polymorphism (PCR-RFLP) as described previously by Bowles and McManus (1993) with some modifications (35). The PCR were performed by using forward and reverse, BD1 (5′-GTCGTAACAAGGTTTCCGTA-3′), 4S (5′-TCTAGATGC GTTCGAA (G/A)TGTCGATG-3′) and EGF1(5′-CCAAACTTGATCATTTAGAGGAAG-3′), EGR2 (5′-TATGG GCC AAATTCACTCATTACC-3) oligonucleotide primers (22, 35).

DNA amplification was performed in a final volume of 25 µl containing 7µl DNA template (200ng/µl), 10 mM Tris-HCl buffer (pH, 9.0), 500 mM KCl, 2.5 mM Mg Cl_2_, 2.5 mM of each dNTP, 15 pmol of each primer (BD1 and 4S, EGF1 and EGR2), 1.5 unit Taq polymerase in reaction buffer. The PCR conditions for each isolates were as follows: an initial denaturing (1 cycle 95 °C for 10 min), followed by 45 cycles denaturation (95 °C for 30s), annealing (57 °C for 1 min), extension (72 °C for 1 min) and final extension (72 °C for 10 min). After amplification the PCR products were electrophoresed through 1% (w/v) Tris-Borate - EDTA (TBE) agarose gels and stained with Ethidum bromide to visualize the separated DNA bands.

The PCR products of each isolates were digested separately for 24 hours (overnight) with three base cutting restriction enzymes of *AluI, MspI* and *TaqI* using 10x assay buffer as recommended by the manufacturer (Sinagen). The digestion by all restriction enzymes were performed by incubating 7µl PCR product with 1.5 µl assay buffer, 6 µl sterile distilled water and 0.5 µl restriction enzymes (8-10 U/ µl) at 37 °C. The DNA fragments were separated by electrophoresis through 3% (w/v) TBE agarose gel (50-100 mV constant voltage). The ethidium bromide stained bands were detected on Gel Doc (Mini-SUB with power Pac Basic, BioRad), and the sizes of PCR products and restriction fragments were analyzed using the UVIdoc images software package.

## Results

### PCR - RFLP analysis

**Fig. 1:**
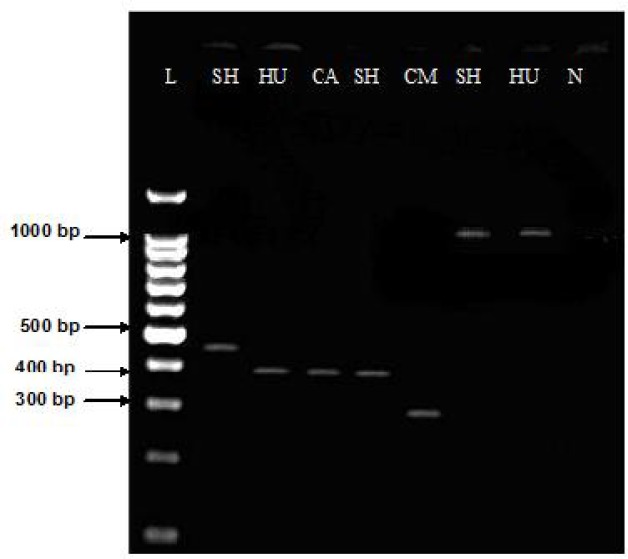
PCR amplified ITS1 fragments from various isolates of *E. granulosus* from Golestan, Iran **SH**: Sheep (size, 1000 bp and 900 bp)/ **HU**: Human (size, 1000 bp and 391) /**CA**: Cattle liver (size, 1391bp)/**CM**: Camel (size, 295 bp)/ **N**: Negative control (without DNA template)/ **L**: DNA lader

In the present study, the region ITS1-PCR and linked ITS1-PCR-RFLP were used to characterize genotypes of *E. granulosus* DNA isolated from hydatid cysts recovered from human,sheep, cattle and camel isolates in the Golestan Province. The ITS1-PCR amplified with BD1 / 4S primers yielded three products of 900 bp, 391 bp and 295 bp and EGF1 / EGR2 primers yielded five products of 1000bp, 900 bp, 471bp, 391 bp and 295 bp in human and animals samples (Table 1). PCR amplification products patterns from sheep, cattle and human isolates yielded unique bands (1000 bp and 391 bp), similar to those obtained with the universal sheep strain. Majority of the camel samples yielded 295 bp PCR products, the products characterized as the camel origin (Table 1. and Fig.1).

**Table 1:** Numbers and sizes of the DNA fragments after PCR amplification with two primers

Primers/ isolates	BD1/ 4S	EGF1/ EGR2
**Sheep**	900 bp	1000 bp
	391 bp	900 bp
		471 bp
		391 bp
**Cattle**	391 bp	1000 bp
	471 bp	391 bp
**Camel**	295bp	471 bp
		391 bp
		295bp
**Human**	391 bp	1000 bp
		391 bp

These results were compared with PCR-RFLP patterns produced after digestion of the ITS1 fragments using restriction endonucleases (*Alu1, Msp1* and *Taq1*). Two clearly distinguishable patterns were obtained with all three enzymes separating isolates from camel origin with those from human, sheep and cattle origin (Table 2).

**Table 2:** Numbers and sizes of the restriction fragments after digestion with different restriction enzymes

Isolates	Restriction endonucleases enzymes		

	*Alu1*	*Msp1*	*Taq1*
	700,300 bp	391 bp*	600,250,150 bp
**Sheep**	900 bp*	–	
	391 bp*	334,137 bp	334,137 bp
	500,400,100 bp	–	–
**Cattle**	334,137 bp	–	
	391 bp*	–	281,110 bp
**Camel**	295 bp*	295 bp*	281,110 bp
**Human**		391 bp*	600,250,150
	391 bp*	281,110 bp	281,110 bp

– No bands were found.

* Show unique bands in a particular isolate as compared to others by same restriction enzyme

**Fig. 2:**
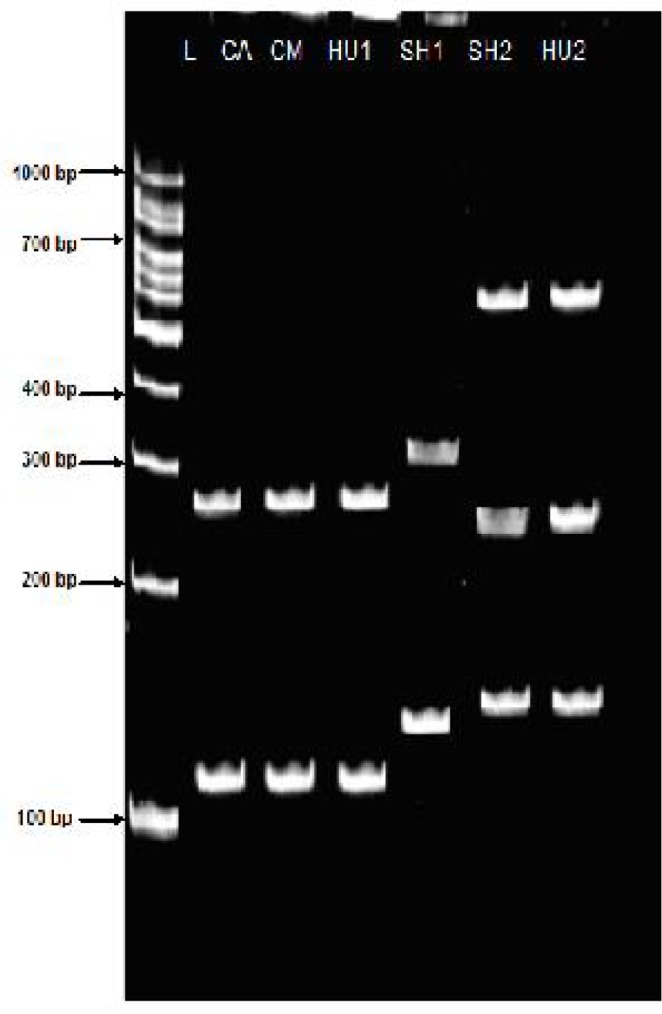
RFLP fragments of various isolates of *E. granulosus* from Golestan, Iran. PCR amplified ITS1 products were digested with ***Taq1***: **SH1**: Sheep (size, 334,137 bp) **SH2**: Sheep (size, 650,250,150 bp) **HU1**: Human (size, 281,110) **HU2**: Human (size, 650,250,150 bp) **CA**: Cattle (size, 281, 110 bp) **CM**: Camel (size, 281,110) **L**: DNA lader

**Fig. 3:**
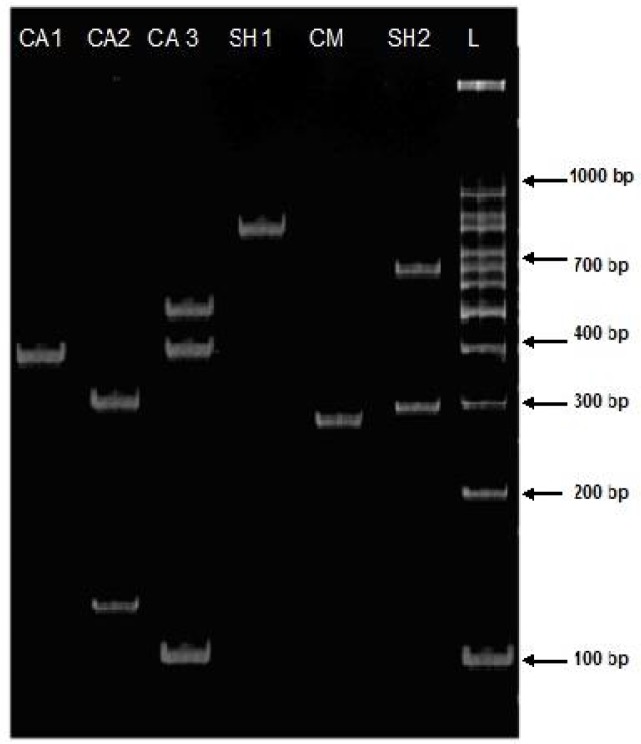
RFLP fragments of various isolates of *E. granulosus* from Golestan, Iran. PCR amplified ITS1 products were digested with ***Alu1***: **CA1**: Cattle (391bp) undigested **CA2**: Cattle (334,137 bp) **CA3**: Cattle (500,400,100 bp) **CM**: Camel (295 bp) undigested **SH1**: Sheep (500 bp) undigested **SH2**: Sheep (700 bp, 300 bp) **L**: DNA lader

*Taq1* restriction enzymes in camel isolate showed a different pattern of genotypes compared with sheep isolates. It could be concluded from the results that sheep isolates are similar to human isolates regarding the banding pattern (Fig. 2 and Table 2). The *Msp1* enzymes reveals 334, 137 bp fragments with identical patterns of DNA in sheep isolates which is similar with the *Taq1* enzyme fragments in sheep and cattle (Table 2 and Fig. 3). Few unique bands of sheep, cattle, camel and human isolates with *Alu1* and *Msp1* were found.

Similarly, *Alu1* breaks the PCR products of sheep and cattle isolates into 2 and 3 fragments respectively; among which, one bands in camel isolates was found distinct from that of sheep isolates. No band with *Alu1* was observed in camel and human isolates (Fig.3, Table 1.). The restriction enzymes, *Msp1* produce identical patterns (bands and size) in sheep and human isolates whereas, no band obtained in cattle and camel isolates (Fig. 3 and Table 1). However, 30 human, 10 sheep and 10 cattle isolates showed PCR products and RFLP patterns of *E. granulosus*, similar to sheep strain, but 10 camel isolates showed two different patters similar to sheep and camel strain (Table 2).

## Discussion

Numerous studies have indicated the occurrence of the *E. granulosus* genotypes (G1-G10) in human and animals (7, 8, 11, 36). In Iran, based on the pervious molecular studies upon sequence variation within the cox1 and nad1 genes, and on PCR-RFLP and PCR-RFLP of the ITS1 region in the nuclear ribosomal gene cluster, the occurrence of G1 and G6 strains of *E. granulosus* in human and different intermediate hosts are indicated (sheep, cattle, camel) (14, 22, 28, 31, 37). Sharbatkhori et al. reported G3 genotype (buffalo strain) in camel from central Iran (30). The workers used different methods like, PCR-RFLP on ITS-1 region of rRNA gene to identify the *E. granulosus* isolates from different hosts in Iran (12, 30, 31, 37). The presence of the sheep and camel strains were also previously demonstrated by Gholami et al. in north of Iran (33).

In the present study, based on ITS1-RFLP patterns of three restriction endonucleases, including *Alu1*, *Msp1* and *Taq1* of the isolates from the tissue samples of human patients infected with hydatid cyst (FFPT) and protoscoleces from hydatid cysts of sheep, cattle and camel isolates, the existence of sheep strain (G1) in human, sheep, cattle, camel the dominant genotype prevailing in Golestan was confirmed. The genotype G1 has been reported in sheep, goat, cattle, camel and human isolates in Iran, whereas, G6 was only determined in camel and in human isolates (12, 21, 25, 28, 30, 33, 34, 37). Sharbatkhori et al. in Isfahan region (Iran), in the camel and human isolates reported the G6 and G1 genotypes (in all of 23 samples of human) and G1 genotype which corresponds our findings (38). In addition, Kia et al. and Sharbatkhori et al. using ITS1-RFLP in six camel isolates in the Isfahan areas found G6 genotype, and in sheep, goat, and cattle isolates in Isfahan and other regions found G1 genotype (23, 39). The presence of G1 as the only genotype in human in these studies and lack of G6 genotype, disagrees with the previous reports on presence of G6 genotype in camels in Isfahan province (14, 23, 38). These researchers speculated that the occurrence of G6 genotype in human in this area is almost rare. Since the sensitivity of different hosts to various *E. granulosus* genotypes in geographical areas differs, genotype identification of cystic echinococcosis in human is significant in managing the of control programs for this zoonotic disease in certain region. Similar studies in other regions of Iran could provide more data on the situation of CE transmission in the country.

The restriction enzymes *Alu1, Msp1* and *Taq1* produced different patterns of the DNA bands in human, sheep, cattle and camel isolates, whereas, similar patterns were observed by *Taq1* in human, camel and cattle. The number and size of the bands obtained by *Taq1* and *Alu1* differed between isolates, indicating that the ITS1 genes of both isolates differ in the number and size. Comparison of the RFLP pattern, obtained by *Taq1* enzymes in all isolates reveal that the 391 bp (cut into 281, 110 bp) fragment was common in cattle, camel and human isolates, whereas, 295 bp fragments was found unique in camel isolates. Further, differences in the numbers and sizes of the bands were also observed between sheep and cattle isolates with *Alu1*. Few unique bands of sheep, cattle, camel and human isolates with *Alu1* and *Msp1* were found. Therefore, on the basis of present findings and the previous reports, it could be suggested that the human, sheep and cattle hydatid cysts are genetically similar. The digestion of the amplified ITS1 fragment of human, sheep, cattle and camel isolates revealed that sheep and camel isolates differ in the banding pattern by *Taq1* and show similar pattern in sheep and cattle by *Alu1* and *Taq1*. However, no bands were observed in camel isolates by *Alu1* and *Msp1*. The restriction enzyme *Taq1* produce exactly similar pattern in human and sheep isolates indicating the homogeneity in the base Paris of rDNA-ITS1 at which this enzyme acts. Similar to the previous study, identical RFLP patterns between horse and cattle strains have been reported after the digestion with *Msp*1 and *Alu1*, however, the other restriction enzymes (*Rsa1*, *CFo* and *Taq1*) produce distinct RFLP patterns (35). Based on apparent conservation of a number of enzyme recognition sites, they suggested that theses two strains are closely related. Fasihi Harandi et al. have shown the similarity in RFLP patterns by *Alu1*, *Msp1* and *Rsa1* restriction enzymes between sheep and camel isolates from Iran (22). Therefore, occurrence of sheep (G1), camel (G6) and buffalo (G3) strains have been demonstrated by mitochondrial gene sequences and PCR-RFLP analysis of rDNA-ITS1 region of a number of isolates from different hosts and different geographical regions of Iran, and the other animals were considered as accidental hosts (28, 29, 31, 34). The results of the present study showed that the G1 strain of *E. granulosus* could be infective for sheep, cattle, camel and human in north of Iran. Therefore, the dominant strain infecting camel might be G1 in Golestan Province (Iran). The base pairs of the fragments obtained in the present study on the sheep, cattle and human isolates were almost similar to that of common sheep strain (*E. granulosus* sensu stricto G1–G3) (5, 10–13, 35). However, the sheep strain is the most common genotype of *E. granulosus*, which affects sheep, cattle, camel and occasionally human (6). Some previous molecular studies in Iran indicated that two distinct cycles (sheep /dog and camel/dog) operate, which overlap and can interact with each other, as infections caused by the sheep and camel strains were detected in camel and sheep respectively as well as in man (12, 29, 31, 34).

In the present work, ITSI –PCR fragments size were observed in hydatid cysts from human isolates (391, 1000 bp), in sheep and cattle isolates (391,471,900, 1000 bp) and camel isolates [295, 391]. Similarly, in pervious study different ITS1 fragments have been reported in the sheep (9.0 kb and 1.0 kb) and camel (1.0 kb and 1.1 kb) isolates of *E. granulosus* from different geographical regions in the world (35). Scott et al. have reported 2 ITS1 fragments common in sheep (9.0 kb and 1.0 kb) and 1 fragment in Polish human isolates (1.04 kb) (10). Kia et al. have found one ITS1 PCR product (approximately 1000 bp) in human isolates and Shahnazi et al. from human and animal (sheep, camel, cattle and goat) reported two amplification products (1.0 and 1.1 kb) of *E. granulosus* in Isfahan ( central of Iran) (23, 24). Bowles et al. reported only one ITS1 PCR product (1.04 kb) in all the 4 isolates of northern cervid form of *E. granulosus* (8). In the present study, the obtained results reveal two different patterns of DNA bands by using primers BD1/4S and EGF1 / EGR2, in camel isolates. Therefore, the results of PCR-ITS1 reveal two different patterns of DNA in camel isolates and similar patterns were observed between human, sheep and cattle isolates. PCR amplification products patterns from sheep, cattle and human isolates were similar to those obtained with the universal sheep strain (G1).

## Conclusion

Comparing the genotypic differences and similarities between *E. granulosus* isolates from human and animals hydatid cysts with PCR-RFLP method identified the occurrence of two genotypes of *E. granulosus* G1(sheep strain) and G6 (camel strain) in Golestan Province (northern Iran). These results reveal the possibility of transmission of the G1 and G6 genotype between livestock animals and human in the north of Iran. Further studies on *E. granulosus* isolates of cattle, camel and other livestock origins are required, which could provide rich data for better understanding about the differences between different cysts localization. The results of the present study on *E. granulosus* genotypes in this area can make a background data for hydatid control programs and warrant the importance of sheep/ dog cycle in public health and considered as a preliminary data for further genetic analyses and local control programs in north of Iran.
